# Diagnostic Accuracy of Interleukin-6, Interleukin-8, and Interleukin-10 for Predicting Bacteremia in Children with Febrile Neutropenia

**DOI:** 10.4274/tjh.2016.0434

**Published:** 2017-08-02

**Authors:** Zümrüt Şahbudak Bal, Nihal Karadaş Özdemir, Semra Şen, Deniz Yılmaz Karapınar, Elif Azarsız, Şöhret Aydemir, Fadıl Vardar

**Affiliations:** 1 Ege University Faculty of Medicine, Department of Pediatrics, Division of Infectious Disease, İzmir, Turkey; 2 Ege University Faculty of Medicine, Department of Pediatrics, Division of Hematology, İzmir, Turkey; 3 Ege University Faculty of Medicine, Department of Pediatrics, Division of Immunology, İzmir, Turkey; 4 Ege University Faculty of Medicine, Department of Clinical Microbiology and Infectious Disease, İzmir, Turkey

**Keywords:** Febrile neutropenia, IL-6, IL-8, IL-10, Bacteremia

## Abstract

Despite improvements in diagnosis and treatment, infections are still a major cause of morbidity and mortality in children with febrile neutropenia. In the majority of febrile episodes, the source of infection cannot be defined. In this study, we aimed to identify the earlier predictors of bacteremia/fungemia and a useful cytokine to identify the source of infection and to discriminate the patients with culture-confirmed bacterial/fungal infection. The most sensitive cytokine was interleukin (IL)-10 and the most specific was IL-8 in predicting culture-confirmed cases. IL-8 had greater sensitivity and specificity in determination of gram-negative bacterial infections with a higher negative predictive value; therefore, IL-8 can be used particularly to rule out gram-negative bacterial infections. IL-6, IL-8, and IL-10 circulating levels were shown to be higher in cases of infection. Further studies are needed to recommend a routine practice for predicting culture-confirmed bacterial infections.

## Abstract (tr)

Tanı ve tedavideki gelişmelere rağmen, febril nötropenili çocuklarda enfeksiyonlar morbidite ve mortalitenin önemli bir nedenidir. Febril nötropeni epizodlarının çoğunluğunda enfeksiyon odağı belirlenememektedir. Bu çalışmada, enfeksiyon odağını ve kültürle kanıtlanmış bakteriyel ve fungal enfeksiyonlarını belirlemede daha erken belirteçler bulunması amaçlanmıştır. Kültür ile kanıtlanmış enfeksiyonları belirlemede en hassas sitokin interlökin (IL)-10 ve en özgül sitokin IL-8’dir. Gram-negatif bakteriyel enfeksiyonları belirlemede en hassas ve özgül sitokin IL-8’dir ve IL-8’in negatif prediktif değerinin yüksek olması nedeniyle, özellikle gram-negatif bakteriyel enfeksiyonları dışlamada kullanılabilir. IL-6, IL-8, IL-10’un kan düzeyleri enfeksiyon durumunda daha yüksek saptanmıştır. Sitokinlerin kültür ile kanıtlanmış enfeksiyonları belirlemede rutin kullanımını önermek için gelecekte yapılacak çalışmalara ihtiyaç vardır.

## INTRODUCTION

In the last few decades, more advanced treatment methods such as myelosuppressive therapy, immunotherapy, and transplantation of hematopoietic stem cells have significantly increased the survival rate of oncologic patients. The development of sepsis and septic shock may be rapid and fatal; therefore, invasive infections require timely and adequate treatment [1]. This study was conducted prospectively to evaluate the potential of interleukin (IL)-6, IL-8, and IL-10 for predicting bacteremia and to compare the levels of IL-6, IL-8, and IL-10 between patients during infection and patients after treatment.

## MATERIALS AND METHODS

Thirty-eight patients (18 females, 20 males) with acute lymphoblastic leukemia (ALL) and acute myeloblastic leukemia (AML) developed 59 febrile neutropenia episodes between June 2014 and March 2015 in this prospective study at the Division of Pediatric Hematology of Ege University Hospital. The median age of the patients was 92.1 months, ranging between 13 and 216 months. Febrile neutropenia was defined according to international guidelines [2]. Medical records were collected including age, sex, diagnosis, and most recent chemotherapy. Laboratory findings, including complete blood count, C-reactive protein (CRP), and bacterial and fungal cultures, were also recorded. Serum samples for analyses of IL-6, IL-8, and IL-10 levels in the blood were collected at the onset (0-24 h) of febrile neutropenia and when patients were afebrile at 72 h after treatment. Serum samples of at least 1 mL were stored at -20 °C until the completion of the study. Levels of IL-6 (ab46042, determination range 1.56-50 pg/mL), IL-8 (ab100575, determination range 0.8-600 pg/mL), and IL-10 (ab46059, determination range 1.56-50 pg/mL) in serum samples were measured using ELISA kits from Abcam (Cambridge, MA, USA) and results were expressed in standardized concentrations using reagents provided with these kits.

### Statistical Analysis

Statistical analyses were performed using MedCalc for Windows (version 15.2, MedCalc Software, Mariakerke, Belgium) and SPSS for Windows (version 22.0, IBM Corp., Armonk, NY, USA). Numerical data were expressed as median (25^th^-75^th^ percentile). Mann-Whitney U and Wilcoxon tests were used for intervariable analysis. Comparisons were referred to as statistically significant at p<0.05. A receiver operating characteristics (ROC) curve was used to determine a cut-off level for the markers; sensitivity and specificity were assessed as equally significant. This study had the permission of the Ethics Board of Ege University (ethical decision number: 13-4.1/12) and written consent was received from all enrolled patients or their parents.

## RESULTS

**Figure 1:**
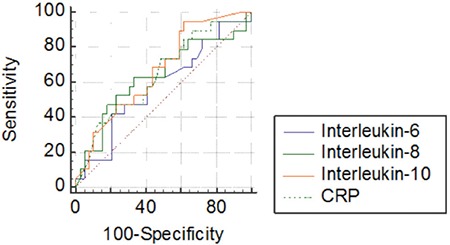
ROC curves of interleukin (IL)-6, IL-8, IL-10, and C-reactive protein in predicting bacteremia. CRP: C-reactive protein.

A total of 59 febrile neutropenia episodes were recorded during the study period. Of the 59 febrile neutropenia episodes, 14 (23.7%) episodes were microbiologically documented by positive blood cultures. The blood cultures revealed gram-positive microorganisms in 5 episodes (5 coagulase-negative Staphylococcus cases), gram-negative microorganismsin 8 episodes (3 Klebsiella pneumoniae, 3 Escherichia coli, and 2 Pseudomonas aeruginosa), and Candida parapsilosis in 1 episode. To compare the values of the groups with and without culture-confirmed infection, ROC curves demonstrating the values of sensitivity, specificity, positive predictive value, and negative predictive value (NPV) for IL-6, IL-8, IL-10, and CRP (Table 1) and the area under the curve (AUC) are shown in Figure 1. The AUC values are summarized in Table 2. In the comparison of the levels of the cytokines, IL-6, IL-10, and CRP were statistically higher in patients with infection than the post-treatment values (Table 3).

## DISCUSSION

In patients with chemotherapy-induced neutropenia, early markers are needed to distinguish the patients at high risk for bacterial or fungal infections that occur independently of the underlying disease. Castagnola et al. [1] evaluated 614 febrile neutropenia episodes and the rate of fever of unknown origin (FUO) was 79%. Kallio et al. [3] evaluated 66 adult patients and reported that CRP, pro-calcitonin, and IL-8 levels were statistically higher in the infection group with 32% sensitivity and 90% specificity for IL-8. IL-8 demonstrated great sensitivity and specificity, particularly in gram-negative bacterial infections, in this study. Miedema et al. [4] reported 52 febrile neutropenic episodes in 32 children; IL-8 was significantly higher in patients with bacteremia and they also determined that the median level of IL-8 was significantly higher in bacteremia caused by gram-negative bacteria than gram-positive bacteria (678 vs.140 ng/L). Similarly, IL-8 demonstrated great sensitivity and specificity particularly in gram-negative bacterial infections in this study. However, IL-8 showed weaker sensitivity when all gram-negative and gram-positive infections were included and this could be a result of the small number of febrile neutropenia episodes included. Urbonas et al. [5] evaluated 61 febrile neutropenia episodes of 37 pediatric patients and reported similar sensitivity and specificity for IL-6 (81%, 75%) and IL-8 (67%, 84%), respectively, but on the second day the sensitivity levels for IL-6 and IL-8 were lower (65%, 61%) than the first day while the specificity values were greater (78%, 89%) for both IL-6 and IL-8, respectively. In this study, sensitivity was found to be lower for IL-6 and IL-8 but both IL-6 and IL-8 had similar specificity. In contrast, IL-10 had 92.9% sensitivity for detecting patients with microbiologically documented infection and the NPV was 95.2; therefore, IL-10 may be a useful marker for ruling out culture-confirmed infection.

Vänskä et al. [6] illustrated the potential role of IL-10 to predict a high risk of complications at the onset of neutropenic fever due to the highest NPV. As they suggested, the AUC demonstrated the best discriminatory power for IL-10 and showed the highest sensitivity for detecting bacterial infections in this study.

Fleischhack et al. [7] suggested that CRP, IL-8, and IL-6 may be less useful than procalcitonin in neutropenic cancer patients, but they only compared gram-negative bacteremia and FUO as primary endpoints. In our study, when only gram-negative bacterial infections were considered, IL-8 was the best marker in discrimination and showed the highest sensitivity and specificity. IL-8 may be useful in detecting gram-negative bacterial infections.

Miedema et al. [8] evaluated 43 pediatric patients as having bacterial infection or not and found that IL-8 was superior to CRP and procalcitonin. IL-8 was more sensitive in predicting bacterial infection at the onset of febrile neutropenia. In our study, we found that IL-8 was a strong predictive marker for bacteremia, particularly for gram-negative bacteremia, as compared to IL-6 and CRP. On the other hand, IL-10 showed greater sensitivity among culture-confirmed bacterial infections.

Diepold et al. [9] found that IL-6 was the best predictor of bacteremia and severe bacterial infection with high sensitivity and specificity (90% and 85%, respectively). In contrast, our data demonstrated better sensitivity and specificity for IL-8 and IL-10 than IL-6.

De Bont et al. [10] reported that IL-6, IL-8, and CRP were significantly higher in patients with gram-negative bacteremia than patients with gram-positive bacteremia, and our data also showed that IL-8 and IL-10 were good at detecting gram-negative bacteremia.

The strengths of this study are it’s prospective design, the relative homogeneity of the patients, and the chance to compare the levels of cytokines during infection and after treatment in the same patients. On the other hand, the current study had limitations including the small number of febrile neutropenia episodes and the lack of the study of genetic polymorphisms. The number of neutropenic fever episodes encountered was relatively low and further studies including larger numbers of patients are needed.

## CONCLUSION

The most sensitive cytokine was IL-10 and the most specific was IL-8 in predicting culture-confirmed infections. IL-8 had greater sensitivity and specificity in determination of gram-negative bacterial infections and a higher NPV; therefore, IL-8 may be used particularly to rule out gram-negative bacterial infections. IL-6, IL-8, and IL-10 circulating levels were shown to be higher during infection and further larger studies are needed to confirm these findings.

## Figures and Tables

**Table 1:**
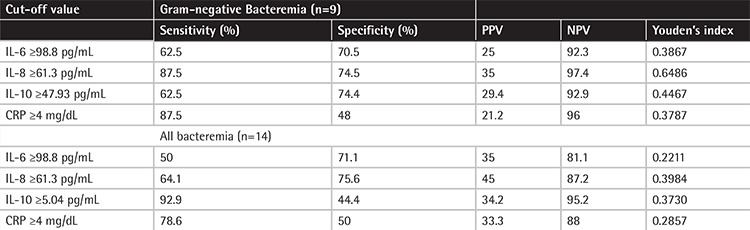
Sensitivity, specificity, positive predictive value, and negative predictive value for interleukin (IL)-6, IL-8, IL-10, and C-reactive protein.

**Table 2:**
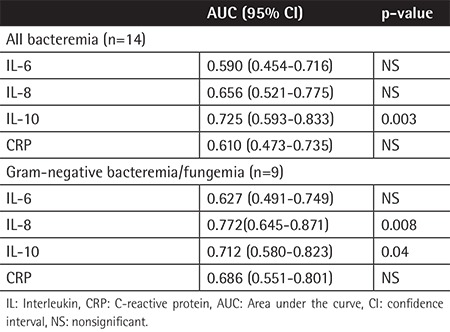
Interleukin (IL)-6, IL-8, IL-10, and C-reactive protein as predictors for bacteremia and gram-negative bacteremia/fungemia (results from receiver operating curve analysis).

**Table 3:**
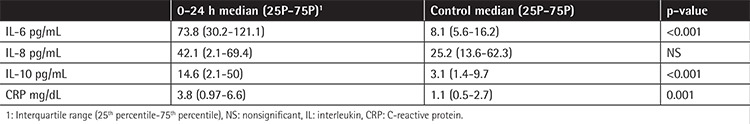
Comparison of the interleukin (IL)-6, IL-8, IL-10, and C-reactive protein levels during infection and after treatment.
